# Isolation and Identification of *Limosilactobacillus reuteri* PSC102 and Evaluation of Its Potential Probiotic, Antioxidant, and Antibacterial Properties

**DOI:** 10.3390/antiox12020238

**Published:** 2023-01-20

**Authors:** Md. Sekendar Ali, Eon-Bee Lee, Suk-Kyung Lim, Kyoungho Suk, Seung-Chun Park

**Affiliations:** 1 Department of Biomedical Science and Department of Pharmacology, School of Medicine, Brain Science and Engineering Institute, Kyungpook National University, Daegu 41944, Republic of Korea; 2 Laboratory of Veterinary Pharmacokinetics and Pharmacodynamics, College of Veterinary Medicine, Kyungpook National University, Daegu 41566, Republic of Korea; 3 Department of Pharmacy, International Islamic University Chittagong, Kumira, Chittagong 4318, Bangladesh; 4 Bacterial Disease Division, Animal and Plant Quarantine Agency, 177 Hyeksin 8-ro, Gimcheon-si 39660, Republic of Korea; 5 Cardiovascular Research Institute, Kyungpook National University, Daegu 41566, Republic of Korea

**Keywords:** *Limosilactobacillus reuteri* PSC102, probiotic properties, antioxidant activity, antibacterial activity

## Abstract

We isolated and characterized *Limosilactobacillus reuteri* PSC102 and evaluated its probiotic, antioxidant, and antibacterial properties. We preliminarily isolated 154 candidates from pig feces and analyzed their Gram nature, morphology, and lactic acid production ability. Based on the results, we selected eight isolates and tested their ability to produce digestive enzymes. Finally, we identified one isolate using 16S rRNA gene sequencing, namely, *L. reuteri* PSC102. We tested its probiotic properties in vitro, including extracellular enzyme activities, low pH and bile salt tolerance, autoaggregation and coaggregation abilities, adhesion to Caco-2 cells, antibiotic susceptibility, and hemolytic and gelatinase activities. Antioxidant activity was determined using 1-diphenyl-2-picrylhydrazyl and 2-azinobis-(3-ethylbenzothiazoline-6-sulfonic acid) diammonium salt radical scavenging and reducing power assays. The antibacterial activity of this strain and its culture supernatant against enterotoxigenic *Escherichia coli* were evaluated using a time-kill assay and disk diffusion method, respectively. *L. reuteri* PSC102 exhibited tolerance toward low pH and bile salt and did not produce harmful enzymes or possess hemolytic and gelatinase activities. Its intact cells and cell-free extract exhibited potential antioxidant activities, and significantly inhibited the growth of enterotoxigenic *E. coli*. Our results demonstrate that *L. reuteri* PSC102 is a potential probiotic candidate for developing functional feed.

## 1. Introduction

Recently, the use of probiotics as growth promoters and alternatives to antibiotics for farm animals, including pigs, has increased significantly [1]. In farm animals, probiotics improve feed utilization efficiency, modulate immunity, and prevent gastrointestinal (GI) infections by developing GI health [2]. Among other probiotics, lactic acid bacteria (LAB) are potential sources of feed supplements for swine nutrition, and are effective for producing functional feeds [3,4,5]. Hence, it is important to evaluate the probiotic features of LAB strains isolated from various sources before utilizing them in functional feed products [6]. Live probiotics must be safe for consumption, capable of surviving in the GI tract, have beneficial characteristics, and be used effectively [7]. Furthermore, they should be able to adhere and multiply in the gut, tolerate bile salt concentrations and gastric acidity, and possess autoaggregation and coaggregation abilities. Moreover, they should not produce any harmful enzymes or exhibit hemolytic and gelatinase activities [8].

LAB possessing antioxidant properties, including free radical scavenging, reducing power, and enzyme inhibition, might ameliorate stress-related disorders. This increases the growth performance of the host by counteracting the reactive oxygen metabolites created during normal cellular processes, such as protein damage, modification of beneficial lipoproteins, mutation of DNA, and oxidation of phospholipids [9,10,11].

Infectious diseases, such as GI infections, are serious threats to swine production as they cause severe illness and death in swine populations [12]. Noninfectious bacteria, such as probiotics, can help maintain mucosal integrity and thus prevent infections by reducing paracellular permeability, defending against pathogens, and increasing the physical barrier of the mucosal layer [13,14]. Probiotic LAB and their culture supernatants can be used as antibacterial components for treating bacterial infections [15]. Previous studies have demonstrated that the production of different antibacterial components by *Lactobacillus*, such as organic acids (acetic and lactic acids), proteinaceous compounds (bacteriocins), and miscellaneous compounds (reuterin), can act as selective barriers against GI pathogens [16,17].

Factors such as the safety profile of probiotics, their survivability in standard GI tract conditions, and other potential benefits must be considered while screening probiotic bacteria [18]. Therefore, this study aimed to isolate, characterize, and identify a new probiotic strain, *L. reuteri* PSC102, and evaluate its potential probiotic, antioxidant, and antibacterial properties.

## 2. Materials and Methods

### 2.1. Bacterial Strains, Culture Conditions, and Media

Different bacterial strains were used in this study. *Escherichia coli* strains (KVCC1423, KVCC0543, and KVCC0306) were provided by the National Veterinary Research and Quarantine Service (Gimcheon, South Korea) [19]. *E. coli* ATCC 35,218, *Bacillus subtilis* KCTC 1021, *Staphylococcus aureus* ATCC 29,213, *Lactobacillus acidophilus* KCTC 3146, and *Limosilactobacillus reuteri* KCTC 3594 were used as quality control strains. All *E. coli* strains were grown in Luria-Bertani (LB) Broth (Difco, Sparks, MD, USA). *L. reuteri* and *L. acidophilus* were cultured in De Man, Rogosa, and Sharpe (MRS) broth (MB Cell, SeoCho-Gu, Seoul, Korea).

### 2.2. Isolation and Selection of L. reuteri PSC102

We collected fecal samples from 80 commercial weaning piglets (Duroc × Landrace × Yorkshire) from breeding farms in Kyungsan city, Gyeonsangbuk province, South Korea. Healthy weaning piglets (weighing 8.5 ± 0.7 kg, aged 4–5 weeks) were not administered any antibiotics or probiotics, and were fed a normal diet. Fecal samples were collected by rectal palpation using sterile swabs. Samples were collected in individual sterile flasks, stored under refrigeration, transported to the laboratory, and processed within 3 h of collection. *Lactobacilli* strains were isolated from the fecal samples as previously described [20,21]. The experiment was exempted from review by the institutional animal care and use committee because it did not involve direct experimentation on the animals.

Briefly, 1 g fecal sample was mixed and homogenized with 9 mL of diluent that consisted of 4.5 g dipotassium hydrogen phosphate (Sigma-Aldrich, St. Louis, MO, USA), 0.5 g L-cystein (Duksan, Daejon, Korea), 0.5 g Tween 80 (Difco, Sparks, MD, USA), and 1 g Bacto agar (Becton, Dickinson and Company, Sparks, MD, USA) in 1 L of distilled water. Then, 1 mL of this solution was serially diluted 10-fold using 0.1% Bacto agar saline. Each diluted solution was streaked on an MRS agar (MB Cell, SeoCho-Gu, Seoul, Korea) plate, followed by anaerobic incubation for 48 h at 37 °C. Each colony was examined for appearance, Gram staining, microscopic cell morphology, catalase generation by H_2_O_2_ (KPL, Gaithersburg, MD, USA), acid production on MRS agar using 0.2% calcium carbonate (Sigma-Aldrich, St. Louis, MO, USA), and lactic acid production using a lactic acid detection kit (Accuvin LLC, Napa, CA, USA). LAB were selected based on their ability to produce dietary enzymes, including protease, lipase, amylase, and phytase, in a modified MRS agar medium (pH 7). Subsequently, they were grown on an MRS agar plate containing 0.2% methyl cellulose and 0.2% corn starch. After 48 h of anaerobic incubation, 0.2% Congo agar red reagent was added to the modified MRS agar. After 30 min, 1 M NaCl was added to decolorize the medium, and the colonies were identified by observing a surrounding halo zone.

To isolate LAB producing a specific enzyme, such as protease, lipase, amylase, and phytase, the colonies were assayed for halo formation on specific media as previously described [20]. Protease-producing colonies were screened by incubating them for 48 h on 1.5% agar medium containing 1% beef extract, 0.5% polypeptide, 0.5% milk casein, and 0.5% NaCl. Lipase-producing colonies were screened by incubating them for 48 h on 1.5% agar medium containing 0.1% tryptone, 0.5% yeast extract, 0.05% NaCl, and 0.1% tricaprylin. Phytase-producing colonies were screened by incubating them for 48 h on 1.5% agar medium containing 1.5% D-glucose, 0.5% calcium phytate, 0.5% NH_4_NO_3_, 0.05% MgSO_4_.7H_2_O, 0.01% MnSO_4_.7H_2_O, 0.05% KCl, 0.001% FeSO_4_.7H_2_O, and 0.01% MnSO_4_.4H_2_O. Amylase-producing colonies were screened by incubating them for 48 h on 1.5% agar medium containing 0.5% polypeptone, 0.5% beef extract, 0.2% yeast extract, 0.2% NaCl, and 2% starch. After 48 h of incubation under anaerobic conditions, the media were examined for colonies with a halo zone. The LAB that could produce all the tested enzymes were subcultured and stored at −70 °C for subsequent analysis.

### 2.3. Identification of L. reuteri PSC102

We identified the strain by analyzing the 16S rRNA gene sequence. Briefly, we extracted the genomic DNA from the isolated strain using a genomic DNA extraction kit (Qiagen Inc., Hilden, Germany) and performed polymerase chain reaction (PCR) using forward and reverse primers (5′-AGGTAACGGCTTACCAAGGC-3′ and 5′-CCACCGCTACACATGGAGTT-3′, respectively). A PCR mixture containing 50 pmole primers, 50 ng template DNA, 5 μL of 10× Taq DNA polymerase buffer, and 1 U of Taq DNA polymerase (Takara, Japan) was denatured at 94 °C for 5 min and then at 95 °C for 30 s. The PCR included 35 cycles with annealing for 30 s at 56 °C, elongation at 72 °C for 30 s, and then final extension for 7 min at 72 °C. BLAST software (version 2.8.1) was used to compare closely related sequences retrieved from the GenBank database.

### 2.4. Scanning Electron Microscopy (SEM) Analysis

SEM was used to observe the morphology of *L. reuteri* PSC102 as previously described [22]. Briefly, *L. reuteri* PSC102 cells were fixed in 2.5% glutaraldehyde in phosphate-buffered saline (PBS) for 2 h and then washed thrice with PBS. After washing, the bacterial cells were dehydrated using graded ethanol concentrations of 30, 50, 70, 80, 90, and 100%. The samples were frozen overnight at −70 °C followed by lyophilization in a vacuum freeze dryer (Operon Advantech Co., Ltd., Gyeonggi, South Korea) for 24 h. The prepared samples were sputter-coated with gold-palladium and analyzed using SEM (S-4300; Hitachi, Tokyo, Japan).

### 2.5. Characterization of L. reuteri PSC102

To establish a culture system, we tested the glycolytic capacity of *L. reuteri* PSC102 using the API 50 CHL kit according to the manufacturer’s instructions (API Biomerieux, Durham, NC, USA). *L. reuteri* PSC102 was suspended in API 50 CHL media, dispensed into strips, and incubated for 48 h at 37 °C. The reading of the strip was determined as negative (−) or positive (+) based on the color change of each tube.

### 2.6. Extracellular Enzyme Activities

We used the API-ZYM kit (Biomeriux, Marcy-I’Etoile, France) to measure the extracellular enzymatic activities according to the manufacturer’s instructions. Briefly, a single colony of *L. reuteri* PSC102 was inoculated in MRS broth and incubated at 37 °C overnight. After centrifugation, the collected cell pellets were mixed with the provided API suspension (0.85% saline), and the turbidity was adjusted with the supplied McFarland 0.5 standard. Next, 65 µL of cell suspension was loaded into each of the 20 cupules of the supplied strip, and then the strip was inserted into the supplied moisture box to prevent drying. After incubation for 4 h at 37 °C, the ZYM-A and ZYM-B reagents were then added dropwise into each cupule. The color changes were observed after 5 min and compared with the manufacturer’s standard response chart. The results were graded from 0 (no activity) to 5 (maximum activity) based on the color intensity per the manufacturer’s instructions.

### 2.7. Acid Tolerance Test

The survivability of *L. reuteri* PSC102 in a low pH milieu was accomplished by measuring the survivable colony counts. Briefly, 1 N HCl was added to 5 mL of MRS medium to adjust the pH to 2, 3, 4, 5, and 7 (control), and then 10^5^ CFU/mL *L. reuteri* PSC102 and *L. reuteri* KCCM 40,717 were added, and growth was confirmed at 0, 1, 6, and 12 h intervals by incubation at 37 °C. The number of viable colonies, expressed as log CFU/mL, was determined by evaluating the sample at designated time points and incubating on MRS agar plates.

### 2.8. Bile Tolerance Test

The resistance of *L. reuteri* PSC102 to bile salts was measured by evaluating its survivability in sterile MRS broth (5 mL) supplemented with different bile salt concentrations (0, 0.1, 0.3, and 1% DifcoTM Oxgall, BD, Franklin Lakes, NJ, SUA) as previously described [23]. Subsequently, *L. reuteri* PSC102 and KCCM 40,717 cells were diluted to 10^5^ CFU/mL and incubated at 37 °C. The number of viable colonies (expressed as log CFU/mL) was determined by taking the samples at 0, 1, 6, and 12 h, followed by incubation on MRS agar plates.

### 2.9. Autoaggregation and Coaggregation Assay

We evaluated the autoaggregation ability of *L. reuteri* PSC102 as previously described, with slight modification [24]. Overnight bacterial culture was harvested by centrifugation (5000× *g*, 4 °C, 10 min) and washed twice with sterile PBS. The pellets were suspended in PBS and the absorbance was maintained at 0.5 ± 0.05 (*OD_initial_*) at 600 nm. The bacterial cell suspension (4 mL) was incubated at 37 °C for different time periods. The percentage of autoaggregation was determined using the following equation:

$$Autoaggregation\;\left(\%\right)=\left(1-\frac{ODtime}{ODinitial}\right)\times 100$$

where *OD_time_* represents the absorbance at 2, 4, 6, 12, or 24 h and *OD_initial_* represents the absorbance at 0 h.

For the coaggregation assay, the bacterial cell suspension (*L. reuteri* PSC102/pathogenic bacteria) was prepared similarly as the autoaggregation assay. OD_600_ of the bacterial suspension was maintained at 0.5 ± 0.05. Equal volumes of *L. reuteri* PSC102 and different pathogenic bacterial cell suspension (2 mL each) were mixed by vortexing for 30 s and incubated for different time periods (2 or 24 h) at 37 °C. The percentage of coaggregation was determined using the following equation:

$$Coaggregation\;\left(\%\right)=\left(1-\frac{ODtime}{ODinitial}\right)\times 100$$

where *OD_time_* represents the absorbance at 2 or 24 h and *OD_initial_* represents that at 0 h.

### 2.10. Adhesion to Human Colon Carcinoma (Caco-2) Cells

The adhesion capacity of *L. reuteri* PSC102 was evaluated using the Caco-2 cell line as previously described [25]. Caco-2 cells were obtained from the Korean Cell Line Bank (Seoul, Korea) and cultured in Roswell Park Memorial Institute (RPMI) medium supplemented with 10% fetal bovine serum albumin and 1% penicillin–streptomycin. The cells were then seeded in a 24-well plate at 10^5^ cells/well and incubated at 37 °C under 5% CO_2_ until they had formed a confluent monolayer. The medium was replaced every alternate day. After the Caco-2 cell monolayer was formed, the media was replaced with antibiotic-free RPMI media. Next, the cell monolayer was washed thrice with PBS before the adhesion assay. *L. reuteri* PSC102 (10^8^ CFU/mL) was added to each well to a final volume of 1 mL, followed by incubation for at 37 °C for 3 h. The wells were washed thrice with sterile PBS to eliminate the non-adhered bacteria. Subsequently, 1 mL of 1% (*v*/*v*) Triton X-100 was added into each well and the mixture was agitated for 10 min to detach the *L. reuteri* PSC102 cells from the wells. The cells were serially diluted 10-fold, streaked on an MRS agar plate, and incubated at 37 °C for 24 h to determine the viable cell count. The percentage adhesion rate of *L. reuteri* PSC102 to Caco-2 cells was determined by dividing the number of adhered bacterial cells (*N*3 *h*) with the initial number of bacterial cells (*No h*) as follows:

Adhesion rate (%)=(N3 h/ No h)×100

### 2.11. Antibiotic Sensitivity Test

The antibiotic sensitivity of *L. reuteri* PSC102 was determined against 14 antibiotics, including cephalexin, colistin sulfate, enrofloxacin, cefalonium, amoxicillin trihydrate, penicillin G procaine, norfloxacin, spectinomycin, tylosin base, cefuroxime sodium, florfenicol, penicillin G benzathine, gentamicin sulfate, and streptomycin sulfate (Sigma-Aldrich, St. Louis, MO, USA), as previously described [26]. Briefly, 100 µL of cultured *L. reuteri* PSC102 was mixed with 100 µL of diluted antibiotic solution in a 96-well plate. After finally adjusting the concentration to 10^6^ CFU/mL, the bacteria were incubated for 24 h at 37 °C. The minimum inhibitory concentration (MIC) and minimum bactericidal concentration (MBC) were determined by measuring the OD value at 600 nm using Gen5 microplate reader version 3.08 (BioTek, Winooski, VT, USA) and by streaking on Mueller–Hinton agar plate, respectively.

### 2.12. Hemolytic and Gelatinase Activities

The hemolytic activity of *L. reuteri* PSC102 was assayed using a blood agar plate (BBL Microbiology Systems, Franklin Lakes, NJ, USA). A single colony of *L. reuteri* PSC102 and *S. aureus* ATCC 29,213 (positive control) were cultured overnight. The cultured bacterial cells were streaked on blood agar plate and incubated for 24 h at 37 °C. Finally, the nonhemolytic activity was evaluated based on the no inhibition zone around the colony [8]. Gelatinase activity was assayed following a previously reported method [27]. Briefly, 10 µL of fresh *L. reuteri* PSC102 and *B. subtilis* KCTC 1021 (positive control) cultures were spotted on nutrient agar (23 g/L) supplemented with gelatin (8 g/L), followed by incubation for 24 h at 37 °C. After incubation, the plates were examined to check for the formation of any opaque halos around the colonies, which indicates gelatinase production.

### 2.13. Antioxidant Activity

#### 2.13.1. Sample Preparation

The antioxidant effects of the cultured *L. reuteri* PSC102 intact cells and their cell-free extracts were measured using a previously described method [8]. Briefly, overnight cultured *L. reuteri* PSC102 was centrifuged (5000× *g*, 10 min, 4 °C) to obtain the intact cells. The collected pellets were washed twice and resuspended in PBS (pH 7.4), and the OD_600_ value was adjusted to 0.4 ± 0.05. The cell-free extracts were prepared by ultrasonic (37 kHz for 30 min) disruption of cultured intact cells. The cells were separated by centrifugation for 10 min at 5000× *g* and 4 °C. The collected supernatants were regarded as cell-free extracts.

#### 2.13.2. 1-Diphenyl-2-Picrylhydrazyl (DPPH) Radical Scavenging Activity

DPPH radical scavenging activity of the intact cells and cell-free extract of *L. reuteri* PSC102 was measured according to a previously simplified method [8]. Briefly, 2 mL of the prepared sample and 2 mL of 0.4 mM DPPH solution were dissolved in methanol and incubated in the dark for 30 min at 37 °C. The reaction mixtures were incubated and centrifuged for 10 min at 5000× *g*, and the absorbance of the supernatant was measured at 517 nm. The control consisted of PBS with DPPH but without the sample. The following formula was used to measure the DPPH radical scavenging activity:

DPPH radical scavenging activity (%)=(ODControl−ODSample/ODControl)×100

#### 2.13.3. 2-Azinobis-(3-Ethylbenzothiazoline-6-Sulfonic acid) Diammonium Salt (ABTS) Radical Scavenging Activity

The ABTS free radical scavenging activity of intact cells and their cell-free extracts was determined based on a previously reported method [8]. The ABTS reagent solution was prepared by mixing 7 mM ABTS stock solution with 7 mM potassium persulfate and kept in the dark overnight at room temperature. The absorbance of the ABTS^+^ solution was adjusted to 0.7 at 734 nm by diluting it with distilled water. Subsequently, 150 µL of the prepared sample solution (intact cells/cell-free extract) was added to 1.35 mL of ABTS^+^ solution and incubated at 37 °C for 10 min. The reaction mixtures were centrifuged at 5000× *g* for 10 min to remove the cells and the absorbance was measured at 734 nm. PBS with ABTS and without the sample was used as the control. The following formula was used to determine the ABTS radical scavenging activity:

ABTS radical scavenging activity (%)=(ODControl−ODSample/ODControl)×100

#### 2.13.4. Reducing Power Activity

The reducing power activity of the intact cells and their cell-free extracts were measured based on a previously reported method [28]. Briefly, 500 µL of 1% potassium ferricyanide and an equal volume of samples were mixed and incubated for 20 min at 50 °C. Then, 500 µL of 10% trichloroacetic acid solution was added, and the mixture was centrifuged at 3000× *g* for 5 min. Finally, after mixing 200 µL of 0.1% FeCl_3_ (Sigma-Aldrich, St. Louis, MO, USA) with 500 µL of the upper liquid layer from the centrifuged mixture, the absorbance was measured at 700 nm.

### 2.14. Determination of Antibacterial Activities

#### 2.14.1. Antibacterial Activity of the *L. reuteri* PSC102 Culture Supernatant

We cultured 1% (*v*/*v*) *L. reuteri* PSC102 in MRS media for 24 h at 37 °C. The supernatant was collected by centrifugation (6000 rpm, 10 min, 4 °C) and filtered using a 0.45-µm-pore-size filter. The filtered supernatant was concentrated using a vacuum evaporator to reduce the volume by 10 times. The antibacterial activities were tested at different concentrations (×10, ×5, and ×1) against three *E. coli* strains (KVCC0306, KVCC0543, and KVCC1423) using the disk diffusion method. Briefly, 100 µL of overnight cultured *E. coli* (10^8^ CFU/mL) were moistened on Mueller–Hinton agar (Becton, Dickinson and Company, Sparks, MD, USA) plates. Subsequently, 6-mm paper disks were soaked with 60 μL of the prepared supernatant sample, dried, and placed on the surface of the bacteria-swabbed plate. A standard ampicillin (10 µg) disk was used as the positive control, and a Mueller–Hinton broth (Becton, Dickinson and Company, Sparks, MD, USA)-treated paper disk was used as the negative control. After overnight incubation at 37 °C, the zone of inhibition was measured using slide calipers and expressed in millimeter (mm).

#### 2.14.2. Time-Kill Assay in Cocultures

The enterotoxigenic *E. coli* strain KVCC0306 was cultured in LB media at 37 °C for 24 h and then adjusted to 10^3^ or 10^5^ CFU/mL. The *L. reuteri* PSC102 culture was adjusted to 10^3^, 10^5^, 10^7^, or 10^9^ CFU/mL and cocultured in tubes containing 10 mL of Iso-sentitest–MRS broth (9:1) at 37 °C for 24 h. The sampling was performed at different time points (0, 1, 6, 12, and 24 h) to determine the viable cell count. Sample aliquots (100 µL) were used to prepare 10-fold serial dilutions and poured onto LB agar plates. The bacterial colonies were enumerated and expressed as log CFU/mL after incubating the plate for 24 h at 37 °C. *E. coli* KVCC0306 inoculated in Iso-sentitest–MRS broth without any treatment was used as the normal control, while colistin (0.1 mg/mL) was used as the positive control.

### 2.15. Statistical Analysis

Statistical significance was determined by one-way analysis of variance using the GraphPad Prism software version 8 (GraphPad Software Inc., San Diego, CA, USA). A *p* value of <0.05 was considered statistically significant.

## 3. Results

### 3.1. Screening and Isolation of L.reuteri PSC102

**Figure 1:**
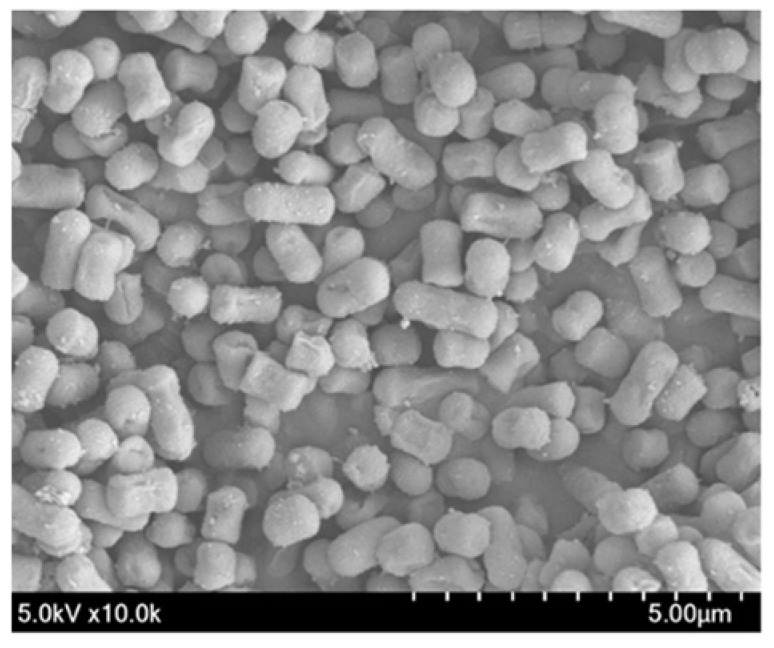
Scanning electron microscope (SEM) image showing the rod-shaped *L. reuteri* PSC102.

A total of 154 (L0001–L0154) candidate strains were initially isolated from pig feces (100 samples). Then, eight *Lactobacilli* strains (L002, L0006, L0010, L0013, L0014, L0017, L0018, and L0102) were selected and phenotypically characterized (Table 1). The strains were identified as gram-positive, rod-shaped, noncatalase forming, and D- and L-lactic acid producers. To identify the potential probiotic LAB candidates, these strains were tested for their ability to produce protease, lipase, amylase, and phytase (Table 2). The strain L0102, i.e., *Limosilactobacillus* (formerly *Lactobacillus*) *reuteri* PSC102 (*L. reuteri* PSC102), was selected as the final probiotic LAB candidate as it produced the maximum amount of protease, lipase, amylase, and phytase. SEM revealed that *L. reuteri* PSC102 had a rod-shaped morphology (Figure 1).

### 3.2. Identification of L. reuteri PSC102

**Figure 2:**
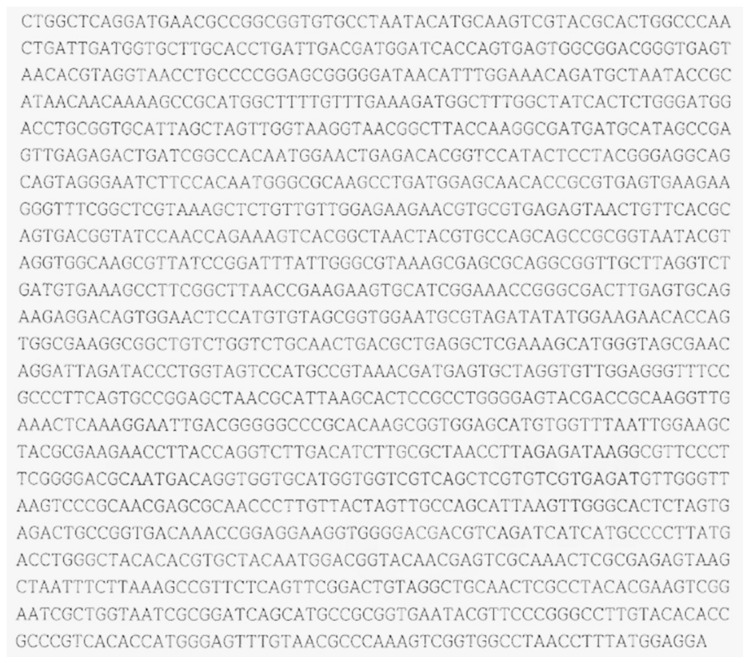
The base sequence of *L. reuteri* PSC102 analyzed using 16S rRNA gene sequencing.

The final selected strain was *L. reuteri* PSC102 (GenBank accession number: MZ127631.1). Figure 2 shows the base sequence analyzed via 16S rRNA gene sequencing (https://www.ncbi.nlm.nih.gov/nuccore/2032707025 (accessed on 12 May 2021). Comparing the GenBank data homology with sequences from the National Center for Biotechnology Information demonstrated that this strain belongs to *L. reuteri* with >99% sequence similarity [22].

### 3.3. Biochemical Characteristics of L. reuteri PSC102

The carbohydrate fermentation profile of *L. reuteri* PSC102 was determined using API 50 CHL medium with API 50 CH strips (Table 3). *L. reuteri* PSC102 exhibited a positive reaction for most carbohydrates, indicating that it could be used as a fermentation starter to produce active metabolites. Therefore, this strain could be efficiently used in industrial media for fermentation based on its glycolytic properties.

### 3.4. Extracellular Enzyme Activities

The extracellular enzymatic profile of *L. reuteri* PSC102 was evaluated using the API-ZYM kit. As shown in Table 4, *L. reuteri* PSC102 showed the best extracellular enzymatic efficacy. The results demonstrated that among the 19 tested enzymes, leucine arylamidase and α-glucosidase were highly produced by the strain, followed by acid phosphatase, naphthol-AS-BI-phosphohydrolase, and α-galactosidase (moderately produced). The production of beneficial enzymes by *L. reuteri* PSC102 was higher than the production of those by the quality control strains *L. reuteri* KCTC 3594 and *L. acidophilus* KCTC 3146.

### 3.5. Acid Resistance of L. reuteri PSC102

To function as a probiotic, the bacterial strain should survive low pH conditions (<pH 3) in the stomach. Therefore, we determined the survivability of *L. reuteri* PSC102 at low pH. As shown in Table 5, *L. reuteri* PSC102 strain survived for >6 h at strongly acidic pH (pH 2 and 3), indicating significantly higher acid resistance compared with that of the standard strain *L. reuteri* KCCM 40,717 (*p* < 0.05).

### 3.6. Bile Salt Tolerance of L. reuteri PSC102

As resistance to bile salts is another important criterion for bacteria to be considered a potential probiotic, we determined the bile salt tolerance of *L. reuteri* PSC102 at different concentrations and time intervals. As shown in Table 6, *L. reuteri* PSC102 could survive even in 1% of bile salt, indicating that this strain possesses excellent bile tolerance. 

### 3.7. Autoaggregation Ability

**Figure 3:**
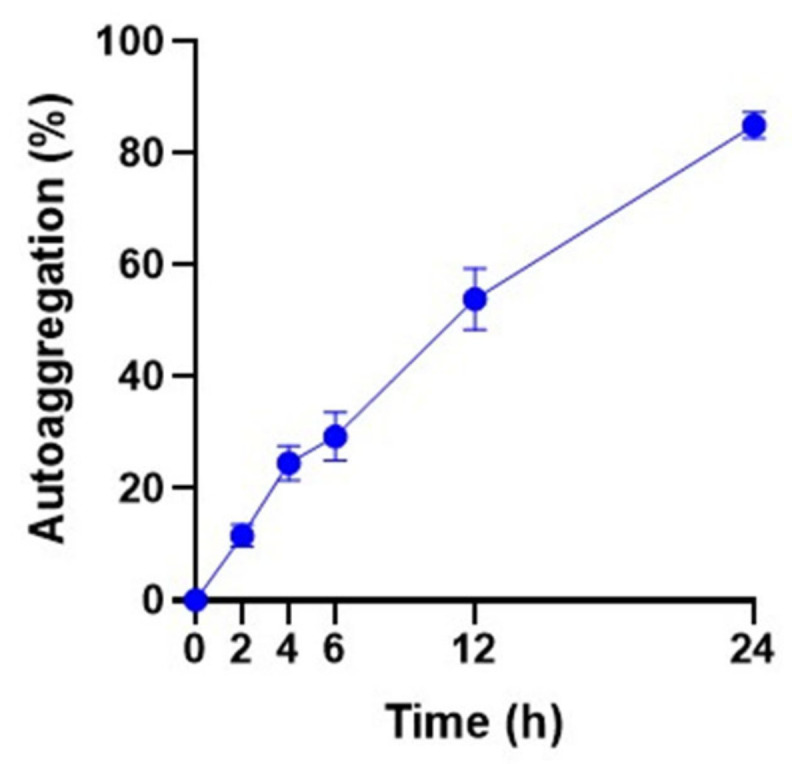
Autoaggregation percentage of *L. reuteri* PSC102 measured at 2, 4, 6, 12, and 24 h of incubation at 37 °C. Data are expressed as mean ± standard deviation (*n* = 3).

**Figure 4:**
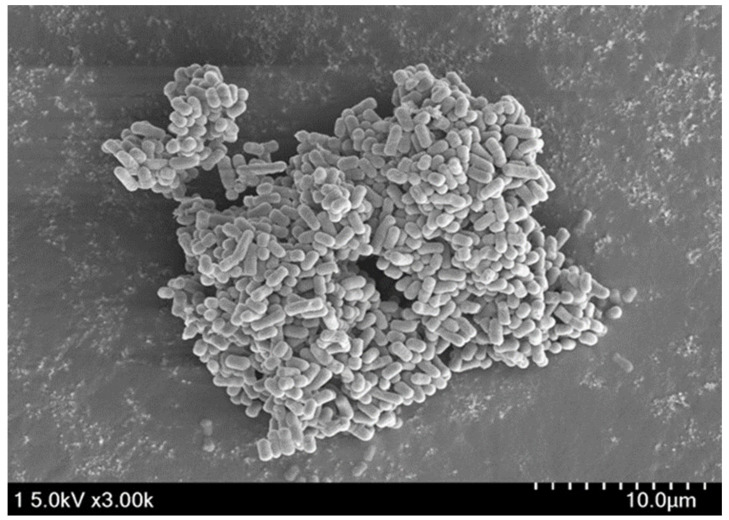
SEM image of autoaggregated *L. reuteri* PSC102.

Autoaggregation was measured over a period of 24 h. The ability of *L. reuteri* PSC102 to autoaggregate increased with increased incubation time (Figure 3 and Figure 4). The strongest autoaggregation ability (84.93%) was observed at 24 h, indicating that *L. reuteri* PSC102 might efficiently adhere to mucosal surfaces and epithelial cells.

### 3.8. Coaggregation Ability

**Figure 5:**
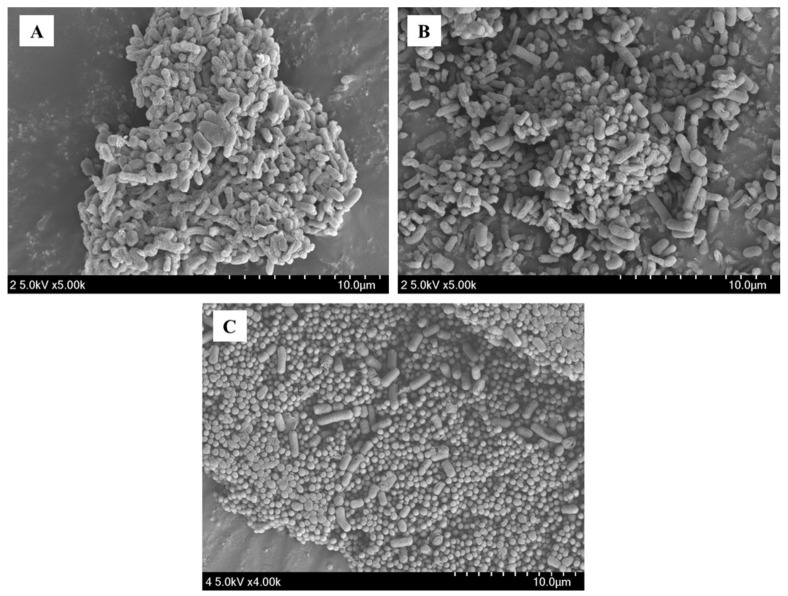
SEM image of *L. reuteri* PSC102 coaggregating with *E. coli* ATCC 35,218 (**A**), *E. coli* KVCC0306 (**B**), and *S. aureus* ATCC 29,213 (**C**).

*L. reuteri* PSC102 was able to coaggregate with pathogens, including enterotoxigenic *E. coli*. After 2 h of incubation, *L. reuteri* PSC102 coaggregated the most with *E. coli* KVCC0306 (9.02%), followed by *S. aureus* ATCC 35,218 (3.35%). After 24 h of incubation, *L. reuteri* PSC102 still coaggregated the most with the same two strains (*E. coli* KVCC0306 [81.13%] and *S. aureus* ATCC 35,218 [72.41%]; Table 7 and Figure 5).

### 3.9. Adhesion to Caco-2 Cells

The initial number of *L. reuteri* PSC102 was 1.14 ± 0.08 (×10^8^) CFU/mL. After 3 h, the number of *L. reuteri* PSC102 adhering to Caco-2 cells was 4.6 ± 0.94 (×10^6^) CFU/mL, with a 4.03% (±0.15) adhesion rate (Table 8). These results are consistent with those of previous studies, which showed that LAB could adhere to Caco-2 cells in the range of 2%–6% [28,29].

### 3.10. Antibiotic Sensitivity Test

Table 9 shows the sensitivity of *L. reuteri* PSC102 to various antibiotics. *L. reuteri* PSC102 strain was found to be resistant to cephalexin, colistin sulfate, norfloxacin, spectinomycin, gentamicin sulfate, and streptomycin sulfate. Moreover, *L. reuteri* PSC102 exhibited higher MIC and MBC values for most of the tested antibiotics than the control strains (*L. reuteri* KCCM 40,717, *S. aureus* ATCC 25,922, and enterotoxigenic *E. coli* KVCC0306) [30,31].

### 3.11. Hemolytic and Gelatinase Activities

**Figure 6:**
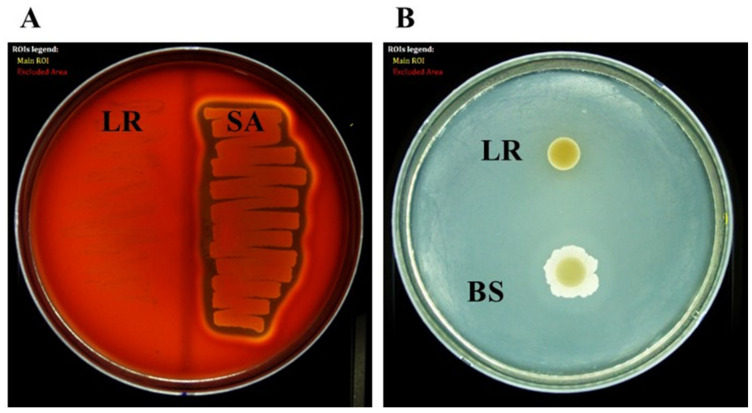
Hemolytic and gelatinase activity test. (**A**) Hemolytic activity: *L. reuteri* PSC102 (LR), positive control; *Staphylococcus aureus* (SA). (**B**) Gelatinase activity: *L. reuteri* PSC102 (LR), positive control; *Bacillus subtilis* (BS).

For the hemolytic activity assay, *L. reuteri* PSC102 was streaked on blood agar plates and incubated at 37 °C for 24 h. We did not observe clear zones (β-hemolysis) or green-hued zones (α-hemolysis) around the colonies (Figure 6A), indicating that *L. reuteri* PSC102 is not a hemolytic strain. Regarding the gelatinase activity, upon checking the plates after incubation, no opaque halos were observed around the colonies (Figure 6B).

### 3.12. Antioxidant Activity

**Figure 7:**
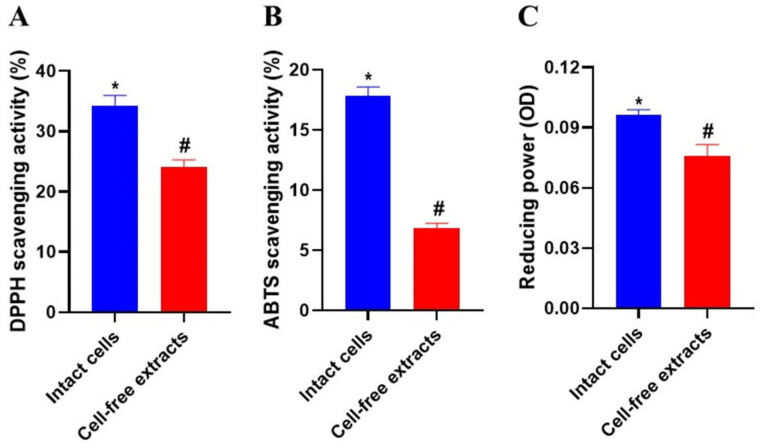
Antioxidant activity of intact cells and cell-free extracts of *L. reuteri* PSC102 measured by (**A**) DPPH radical scavenging, (**B**) ABTS radical scavenging, and (**C**) reducing power assays. Different symbols (*, **^#^**) on the bars denote significant differences between groups (*p* < 0.05). Data are expressed as mean ± standard deviation (*n* = 3).

Figure 7 shows the DPPH and ABTS free radical scavenging activities of *L. reuteri* PSC102 intact cells and cell-free extracts. Intact *L. reuteri* PSC102 cells had higher DPPH radical-scavenging ability (34.31%) than the intracellular cell-free extracts (24.04%; Figure 7A). The ABTS radical scavenging effect of intact cells was 17.84%, which was more than double that of the intracellular cell-free extracts (6.86%; Figure 7B). The reducing power of the intact cells was 0.096, whereas that of the cell-free extracts was 0.076 (Figure 7C). The DPPH and ABTS free radical scavenging activities and reducing power of intact cells were higher than those of the cell-free extracts, indicating that more active components were present in the intact cells.

### 3.13. Antibacterial Activities of L. reuteri PSC102 Culture Supernatant

Evaluation of the potential antibacterial activities of the supernatants of *L. reuteri* PSC102 against three enterotoxigenic *E. coli* strains revealed that the supernatant possessed antibacterial activities against all three *E. coli* strains (Table 10). Among them, *E. coli* KVCC1423 was the most sensitive to ×10 concentrated supernatant (14.72-mm inhibition zone). Interestingly, *E. coli* KVCC0306 was resistant to standard ampicillin but was sensitive to the supernatant.

### 3.14. Time-Kill Assay

**Figure 8:**
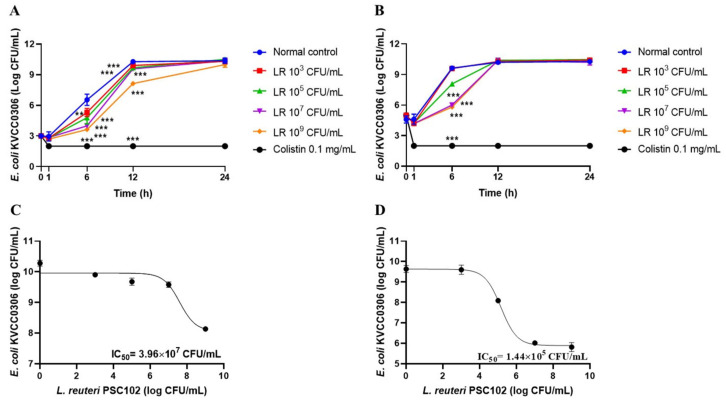
Time-kill assay curves of coculture of *L. reuteri* PSC102 (LR) and enterotoxigenic pathogen *E. coli* KVCC0306. CFU changes of 10^3^ CFU/mL (**A**) and 10^5^ CFU/mL *E. coli* KVCC0306 (**B**) observed after coculture with four different concentrations of *L. reuteri* PSC102 (10^3^, 10^5^, 10^7^, and 10^9^ CFU/mL) for 24 h. The minimum concentration of *L. reuteri* PSC102 needed to inhibit the growth of *E. coli* KVCC0306 by 50% (IC_50_) when *E. coli* KVCC0306 cocultured at 10^3^ CFU/mL (**C**) and 10^5^ CFU/mL (**D**). Data are expressed as means ± standard deviation (*n* = 3). ** *p* < 0.01, and *** *p* < 0.001 vs. normal control (*E. coli* KVCC0306 10^3^ CFU/mL or 10^5^ CFU/mL culture alone).

Figure 8 shows the results of the time-kill assay. After 24 h incubation, the growth of *E. coli* KVCC0306 did not change significantly. However, at 6 and 12 h, the growth of *E. coli* KVCC0306 (10^3^ CFU/mL) was significantly inhibited by *L. reuteri* PSC102 (10^3^, 10^5^, 10^7^, or 10^9^ CFU/mL) compared with the normal control (*p* < 0.05; Figure 8A). With coculture of 10^5^ CFU/mL *E. coli* KVCC0306 and different concentrations of *L. reuteri* PSC102, *E. coli* KVCC0306 was significantly inhibited by *L. reuteri* PSC102 at 6 h (*p* < 0.05; Figure 8B). The CFU/mL of *E. coli* KVCC0306 in the presence or absence of different concentrations of *L. reuteri* PSC102 was adjusted with the inhibitor vs. response model generated using the GraphPad Prism 8 software with the following equation:

Y = Bottom + ((Top − Bottom)/(1 + 10^(X − LogIC_50_)))

where Top and Bottom are *E. coli* KVCC0306 CFU/mL in the absence of *L. reuteri* PSC102 and at maximum growth inhibition in the presence of *L. reuteri* PSC102, and IC_50_ is the minimum concentration of *L. reuteri* PSC102 needed to inhibit the growth of *E. coli* KVCC0306 by 50% in their coculture. For *E. coli* KVCC0306 (10^3^ and 10^5^ CFU/mL), the IC_50_ values of *L. reuteri* PSC102 were found to be 3.96 × 10^7^ and 1.44 × 10^5^ CFU/mL, respectively (Figure 8C,D).

## 4. Discussion

Probiotics are obtained after in vitro screening for evaluating several characteristics, such as the ability to produce digestive enzymes, inhibit various pathogens, tolerate bile salts and gastric acids, exhibit antimicrobial sensitivity, and provide safe and beneficial properties for the host [21,32]. In this study, *Lactobacillus* strains were initially isolated, and their phenotypic characteristics were evaluated. Thereafter, their ability to produce digestive enzymes, including lipase, phytase, amylase, and protease, was examined. We isolated 154 potential *Lactobacillus* probiotics from pig fecal samples and cultured them on MRS media. Further, we identified eight candidates based on their phenotypic characteristics and capacity to produce digestive enzymes. Finally, we identified *L. reuteri* PSC102 based on its ability to produce digestive enzymes. Biochemical evaluation of *L. reuteri* PSC102 using API 50 CHL showed that *L. reuteri* PSC102 possesses glycolytic capacity, suggesting that this strain can be successfully used for fermentation.

Probiotic bacteria have several health benefits for humans and other animals [33,34,35,36,37]. These bacteria should not produce enzymes such as β-glucuronidase, α-chymotrypsin, and N-acetyl-glucosaminidase, as these enzymes are potentially harmful [38]. As expected, *L. reuteri* PSC102 did not produce any harmful enzymes. Conversely, acid phosphatase, α-glucosidase, and β-galactosidase released by probiotics have been shown to be beneficial [39]. In particular, acid phosphatase, when used as a supplementary feed additive, increases the absorption of phosphorus in the feed, thereby reducing the use of inorganic phosphorus.

Probiotic strains must be able to survive in conditions similar to those found in the GI tract, by exhibiting tolerance to bile salts and low pH [40]. In addition to resisting low pH and changes in glycolytic flux, probiotic bacteria should maintain intracellular pH [41]. Low pH can increase the ammonium output in the cytoplasm, which is likely liberated during the deamination of amino acid. This reduces the activity of bile salt hydrolase [41]. H^+^-ATPase activity is also necessary for survival in an acidic environment [8]. Under acidic conditions, the H^+^-ATPase activity of acid-tolerant bacterial strains increases, whereas that of non-acid-tolerant strains decreases [42]. The H^+^-ATPase activity in the cells increases rapidly to sustain a stable intracellular pH by releasing H^+^ for acid-tolerant bacteria [42]. Tolerance to bile salts is important for *Lactobacillus* to grow, survive, and function during GI tract transit [43]. The most common mechanisms of bile salt tolerance in LAB are active efflux and hydrolysis of bile salts and alterations of the cell membrane and cell wall composition [44]. A previous study showed that different *Lactobacillus* species isolated from goat’s milk cheese can hydrolyze bile salts [7]. Interestingly, our tested *L. reuteri* PSC102 strain was resistant to low pH and high bile salt concentration, suggesting that this strain would likely survive the stomach passage and in the small intestine.

Autoaggregation and coaggregation abilities of bacteria are essential for several biological activities [45]. Via aggregation, bacteria may gain sufficient mass to form biofilms or adhere to the host’s intestinal mucosal surfaces, allowing them to perform their functions [46]. In our study, the autoaggregation of *L. reuteri* PSC102 increased with an increase in the incubation period. In a previous study, *Lactobacilli* autoaggregation was facilitated by proteins found in the culture supernatant and lipoproteins or proteins on the surface of the cells that were not washed off and resuspended in PBS [47]. Coaggregation enables bacteria to interact intimately with other bacteria [48]. In this study, the percentage of coaggregation of *L. reuteri* PSC102 with *E. coli* KVCC0306 was the highest after 2 and 24 h of incubation. This ability to coaggregate with pathogens might contribute to the probiotic potential of *L. reuteri* PSC102 by allowing it to establish a barrier and prevent the colonization of harmful bacteria [49,50].

The ability of probiotic bacteria to adhere to the intestinal epithelium mucosa and colonize is a significant characteristic as it prevents removal from the intestine by peristalsis [25,51]. In this study, the ability of *L. reuteri* PSC102 to adhere to intestinal mucosa was evaluated using Caco-2 cells, which proved that it could colonize in the intestine and maintain the intestinal microflora homeostasis of the host.

The functional qualities of probiotic strains should be assessed before in vivo administration. Antibiotic resistance is considered detrimental to human and animal health and food safety [28]. However, the antibiotic susceptibility of a probiotic is a critical factor in determining whether it can be coadministered with antibiotics [52]. In this study, LAB strains exhibited antibiotic reactivity, which is consistent with that reported by a previous study [53]. As *L. reuteri* PSC102 was resistant to the tested antibiotics, it can be used in combination with antibiotic-containing feed or feed additives and exert its probiotic effect. Moreover, in the presence of these antibiotics, *L. reuteri* PSC102 might survive and exert useful effects in the host [54].

As most probiotic bacteria are safe for consumption, the chances of infections are rare. However, hemolysis might occur if the ingested bacteria gains access to the blood, resulting in hemolytic symptoms, including fever, anemia, and skin rash [55]. Therefore, the hemolytic activity of probiotics must be assessed to guarantee their safety. Gelatinase activity is considered a risk factor as it indicates the potential to hydrolyze collagen, which may trigger an inflammatory reaction [56]. In this study, *L. reuteri* PSC102 did not show hemolytic and gelatinase activities, thus eliminating the safety issues regarding this strain.

Oxidative stress can harm cells by initiating DNA hydroxylation, lipid peroxidation, and protein denaturation. However, antioxidants can prevent or reduce oxidative damage [57]. LAB exert antioxidant effects by producing various active cell surface components, proteins, and antioxidant enzymes. This prevents or hinders the progress of different oxidative stress-related disorders [58]. LAB have been shown to possess strong DPPH and ABTS free radical scavenging activity, which might enhance the oxidative status of weaned piglets and improve their growth [59]. In our study, *L. reuteri* PSC102 demonstrated a strong free radical scavenging effect and reducing power. Hence, it might be potentially used to alleviate oxidative stress-induced disorders.

The growth-inhibitory activity of probiotics against target pathogens is a desirable property [60]. Our findings demonstrated that *L. reuteri* PSC102 could inhibit all the tested enterotoxigenic *E. coli* pathogenic strains. *L. reuteri* PSC102 cells and culture supernatant exhibited strong antibacterial activity against all three *E. coli* strains in a concentration-dependent manner. Despite *E. coli* KVCC0306 being resistant to standard ampicillin, the supernatant exhibited inhibitory activity against it. Therefore, we selected *E. coli* KVCC0306 for the time-kill assay by coculturing with live *L. reuteri* PSC102. The supernatant might contain different active metabolites with antimicrobial properties, such as organic acids (mainly lactic and acetic acids) and other metabolites, including p-coumaric, 3-phenylpropanoic, 3-phenyl lactic, D-glucuronic, and benzoic acids; cyclic dipeptides; and bacteriocins, all of which are potent antibacterial compounds [8,61]. The antibacterial activity of the supernatant is mediated by the rapid diffusion of the antimicrobial components across the microbial cell membrane. This activity significantly depends on the concentration of antimicrobial components and the number of pathogenic bacteria used for the assay [8]. The most notable metabolite produced by *L. reuteri* is reuterin, a broad-spectrum antimicrobial component that can kill many food-borne pathogens, including *E. coli* and *S. aureus* [62,63]. Rueterin exerts its activity by getting adsorbed by sensitive bacterial cells and disturbing their metabolism by depleting free sulfhydryl groups in bacterial proteins, thereby inducing oxidative stress and ultimately resulting in cell death [64]. Moreover, lactic and acetic acid production can lower the pH inside the cells, which can dissipate membrane function. The acidification of the cytoplasm might restrict bacterial growth by limiting glycolysis [65]. In the time-kill assay, cocultures of *L. reuteri* PSC102 with the enterotoxigenic pathogen *E. coli* inhibited the growth of *E. coli* KVCC0306 for up to 12 h. During coculture, different organic compounds were produced, which might permeabilize the outer membrane of gram-negative bacteria, such as *E. coli,* and enhance the activities of other antibacterial metabolites [66]. Therefore, this *L. reuteri* strain might effectively treat enterotoxigenic *E. coli*-induced diarrhea in pigs.

## 5. Conclusions

In this study, we isolated and identified *L. reuteri* PSC102, which showed excellent characteristics, including autoaggregation and coaggregation, adhesion to Caco-2 cells, and resistance to in vitro GI tract conditions. The strain did not exhibit any hemolytic and gelatinase activity or produce any undesirable extracellular enzymes. The intact *L. reuteri* PSC102 cells and their cell-free extracts showed promising antioxidant activities. Moreover, both the strain and its supernatant exhibited antibacterial activities against enterotoxigenic *E. coli*. These results suggest that *L. reuteri* PSC102 is an efficient probiotic candidate that can aid in the development of functional feeds. As this strain has been isolated from swine, it could be preferentially used in swine as a growth promoter and an antibiotic alternative.

## Figures and Tables

**Table 1:** Characterization of *Lactobacilli* strains isolated from pig fecal samples based on Gram staining, cell morphology, and acid production.

Isolates	Gram Staining	Cell Morphology	Catalase	Acid Production	Lactic Acid Production
L0002	+	Rod	−	+	D, L
L0006	+	Rod	−	+	D, L
L0010	+	Rod	−	+	D, L
L0013	+	Rod	−	+	D, L
L0014	+	Rod	−	+	D, L
L0017	+	Rod	−	+	D, L
L0018	+	Rod	−	+	D, L
L0102	+	Rod	−	+	D, L

+, Positive; −, Negative.

**Table 2:** Enzymatic activities of the screened *Lactobacilli* strains.

Isolates	Protease	Lipase	Amylase	Phytase
L0002	++	+	+	+
L0006	+++	+	+	++
L0010	++	−	++	−
L0013	++	−	+	+
L0014	+++	−	+	+
L0017	+++	+	+++	++
L0018	++	+	+++	+
L0102	+++	++	+++	+++

+++, Maximum; ++, Medium; +, Minimum; −, Negative.

**Table 3:** Carbohydrate fermentation profile of *L. reuteri* PSC102 using API 50 CHL.

Active Ingredient	Result	Active Ingredient	Result	Active Ingredient	Result
Control	−	Inositol	−	Inulin	+
Glycerol	+	Mannitol	+	Melezitose	−
Erythritol	−	Sorbitol	+	Raffinose	+
D-arabinose	−	α-Methyl-D-mannoside	+	Starch	+
L-arabinose	−	β-Methyl-xyloside	−	Glycogen	+
D-ribose	+	α-Methyl-D-glucoside	−	Xylitol	−
D-xylose	+	N-Acetyl-glucosamine	−	Gentiobiose	−
L-xylose	+	Amygdalin	+	D-Turanose	+
D-adonitol	−	Arbutine	+	D-Lyxose	−
Methyl-β-D-xylopyranoside	−	Esculine	+	D-Tagatose	−
D-galactose	+	Salicine	+	D-fucose	+
D-glucose	+	Cellobiose	+	L-fucose	−
D-fructose	+	Maltose	+	D-arabitol	−
D-mannose	+	Lactose	+	L-arabitol	−
L-sorbose	+	Melibiose	+	Gluconate	−
Rhamnose	−	Sucrose	+	2-keto-Gluconate	+
Dulcitol	−	Trehalose	+		

+, Positive; −, Negative.

**Table 4:** Enzymatic activities of *L. reuteri* PSC102 as determined using the API-ZYM kit.

No.	Enzymes	*Limosilactobacillus reuteri* PSC102	*Limosilactobacillus reuteri* KCTC 3594	*Lactobacillus acidophilus* KCTC 3146
1	Control	0	0	0
2	Alkaline phosphatase	0	0	0
3	Esterase	3	2	2
4	Esterase lipase	1	1	1
5	Lipase	0	0	0
6	Leucine arylamides	5	5	4
7	Valine arylamides	1	3	1
8	Cystine arylamides	1	0	0
9	Trypsin	0	0	0
10	α-Chymotrypsin	0	0	0
11	Acid phosphatase	4	2	2
12	Naphthol-AS-BI-phosphohydrolase	4	2	2
13	α-Galactosidase	4	4	1
14	β-Galactosidase	1	5	4
15	β-Glucuronidase	0	0	0
16	α-Glucosidase	5	2	1
17	β-Glucosidase	0	0	0
18	N-acyl-glucosaminidase	0	0	0
19	α-Mannosidase	0	0	0
20	α-Fructosidase	0	0	0

**Table 5:** Tolerance of *L. reuteri* PSC102 to low pH.

Bacteria	pH 2				pH 3				pH 5				pH 7			
	0 h	1 h	6 h	12 h	0 h	1 h	6 h	12 h	0 h	1 h	6 h	12 h	0 h	1 h	6 h	12 h
	Log CFU/mL				Log CFU/mL				Log CFU/mL				Log CFU/mL			
*L. reuteri* PSC102	5.55 ± 0.08	3.75 ± 0.16 *	3.52 ± 0.09 *	2.00 ± 0.00	5.55 ± 0.08	4.81 ± 0.12	3.74 ± 0.08 *	2.00 ± 0.00	5.55 ± 0.08	4.95 ± 0.05	4.52 ± 0.08 *	7.65 ± 0.14 *	5.55 ± 0.08	5.97 ± 0.06	6.52 ± 0.23	10.75 ± 0.15 *
*L. reuteri* KCCM 40,717	5.29 ± 0.16	2.00 ± 0.00 **^#^**	2.00 ± 0.00 **^#^**	2.00 ± 0.00	5.29 ± 0.16	4.35 ± 0.24	2.00 ± 0.00 **^#^**	2.00 ± 0.00	5.29 ± 0.16	4.94 ± 0.05	5.13 ± 0.11 **^#^**	5.16 ± 0.07 **^#^**	5.29 ± 0.16	5.99 ± 0.15	6.51 ± 0.05	9.17 ± 0.07 **^#^**

Data are expressed as mean ± standard deviation (*n* = 3). Different symbols (*, **^#^**) above the values denote significant differences (*p* < 0.05).

**Table 6:** Bile salt tolerance of *L. reuteri* PSC102.

Bacteria	Bile Salt (0.1%)				Bile Salt (0.3%)				Bile Salt (1%)				Bile Salt (0%)			
	0 h	1 h	6 h	12 h	0 h	1 h	6 h	12 h	0 h	1 h	6 h	12 h	0 h	1 h	6 h	12 h
	Log CFU/mL				Log CFU/mL				Log CFU/mL				Log CFU/mL			
*L. reuteri* PSC102	5.23 ± 0.04	5.27 ± 0.09	5.41 ± 0.11	7.28 ± 0.09	5.23 ± 0.09	5.26 ± 0.02	5.38 ± 0.01	6.83 ± 0.08 *	5.23 ± 0.04	5.20 ± 0.06	5.32 ± 0.06	6.53 ± 0.09 *	5.23 ± 0.04	5.36 ± 0.11	6.65 ± 0.08	9.19 ± 0.09
*L. reuteri* KCCM 40,717	5.19 ± 0.10	5.22 ± 0.08	5.53 ± 0.10	7.27 ± 0.11	5.19 ± 0.05	5.25 ± 0.06	5.37 ± 0.04	6.28 ± 0.06 **^#^**	5.19 ± 0.10	5.17 ± 0.06	5.28 ± 0.04	5.18 ± 0.09 **^#^**	5.19 ± 0.10	5.75 ± 0.06	6.53 ± 0.06	9.09 ± 0.07

Data are expressed as mean ± standard deviation (*n* = 3). Different symbols (*, **^#^**) above the values denote significant differences (*p* < 0.05).

**Table 7:** Coaggregation percentage of *L. reuteri* PSC102 with different pathogenic bacteria as measured after 2 and 24 h of incubation at 37 °C.

Pathogenic Bacteria	Coaggregation with *L. reuteri* PSC102 (%)	
	2 h	24 h
*E. coli* ATCC 35,218	6.52 ± 0.75	77.16 ± 1.59
*E. coli* KVCC0306	9.02 ± 0.91	81.13 ± 0.87
*S. aureus* ATCC 29,213	3.35 ± 0.51	72.41 ± 0.69

Data are expressed as mean ± standard deviation (*n* = 3).

**Table 8:** Adhesion of *L. reuteri* PSC102 to Caco-2 cells.

Initial Concentration (*L. reuteri* PSC102, CFU/mL)	Final Concentration (*L. reuteri* PSC102, CFU/mL)	Adhesion (%)
1.08 × 10^8^	4 × 10^6^	3.70
1.26 × 10^8^	6 × 10^6^	4.76
1.10 × 10^8^	4 × 10^6^	3.63
Average		
1.14 × 10^8^	4.6 × 10^6^	4.03

**Table 9:** Minimal inhibitory concentration (MIC) and minimum bactericidal concentration (MBC) of *L. reuteri* PSC102 against various antibiotics.

Antibiotics	*Limosilactobacillus reuteri* PSC102		*Limosilactobacillus reuteri* KCCM 40,717		*Staphylococcus aureus* ATCC 25,922		Enterotoxigenic *E. coli* KVCC0306	
	MIC (μg/mL)	MBC (μg/mL)	MIC (μg/mL)	MBC (μg/mL)	MIC (μg/mL)	MBC (μg /mL)	MIC (μg/mL)	MBC (μg/mL)
Cephalexin	>256	>256	>256	>256	2	4	32	64
Colistin sulfate	>64	>128	>64	>128	>64	>128	0.05	1
Enrofloxacin	8	16	4	8	0.05	1	0.025	1
Cefalonium	32	64	64	32	2	4	16	32
Amoxicillin trihydrate	4	2	1	1	0.5	2	1	2
Penicillin G procaine	32	64	16	64	0.05	1	32	64
Norfloxacin	>256	>256	>256	>256	0.5	4	0.5	1
Spectinomycin	64	128	8	128	8	64	2	4
Tylosin base	4	8	2	4	1	8	32	64
Cefuroxime sodium	2	32	1	8	0.05	2	1	2
Florfenicol	2	16	2	16	2	8	4	16
Penicillin G benzathine	4	8	2	4	0.05	2	16	32
Gentamicin sulfate	64	128	32	128	4	16	1	2
Streptomycin sulfate	64	128	32	128	4	16	1	2

**Table 10:** Antibacterial activities of *L. reuteri* PSC102 supernatant against enterotoxigenic pathogens evaluated using the disk diffusion method.

Pathogens	Zone of Inhibition (mm)			
	Concentrated Supernatant of *L. reuteri* PSC102			
	×10	×5	×1	Positive Control (Ampicillin, 10 µg)
*E. coli* KVCC0306	14.10 ± 0.08	8.96 ± 0.03	-	-
*E. coli* KVCC0543	14.20 ± 0.06	8.94 ± 0.04	-	20.35 ± 0.35
*E. coli* KVCC1423	16.17 ± 0.51	9.60 ± 0.41	-	21.47 ± 0.65

Data are expressed as means ± standard deviation (*n* = 3). “-“ indicates no inhibition.

## Data Availability

All data generated for this study are contained within the article.
